# A Patient-Reported Outcome Measure of Communication Difficulties in Friedreich Ataxia: COMATAX.

**DOI:** 10.1007/s12311-026-02020-3

**Published:** 2026-05-23

**Authors:** Maresa Buchholz, Victoire Monier, Claire Ewenczyk, Anna Heinzmann, Lucie Pierron, Sabrina Sayah, Rania Hilab, Mariana Atencio, Elodie Petit, Fanny Bertrand, Alix Vallancien, Chloé Diot, Stéphan Rouillon, Andreas Nadke, Katrin Feldmann, Vivian Maas, Jennifer Faber, Dorota Sarwinska, Sylvia M. Boesch, Elisabetta Indelicato, Almut T. Bischoff, Thomas Klopstock, Jörg B. Schulz, Kathrin Reetz, Zofia Fleszar, Audrey Iskandar, Thomas Klockgether, Marcus Grobe-Einsler, Feng Xie, Brittany Humphries, Bernhard Michalowsky, Alexandra Durr, Stéphanie Borel

**Affiliations:** 1 German Center for Neurodegenerative Diseases, Patient-reported Outcomes & Health Economics Research, Site Rostock/Greifswald, Greifswald, Germany; 2 Sorbonne Université, Institut du Cerveau-Paris Brain Institute-ICM, CNRS, Inserm, AP-HP, Groupe Hospitalier Sorbonne Université, Paris, France; 3 Association Française de l’Ataxie de Friedreich, Hirson, France; 4 Deutsche Heredo-Ataxie-Gesellschaft, Stuttgart, Germany; 5 German Center for Neurodegenerative Diseases, Bonn, Germany; 6 Department of Parkinson Disease, Sleep and Movement Disorders, Center for Neurology, University Hospital Bonn, Bonn, Germany; 7 Center for rare Movement Disorders Innsbruck, Department of Neurology, Medical University Innsbruck, Innsbruck, Austria; 8 Department of Neurology, Friedrich-Baur-Institute, LMU University Hospital, Ludwig- Maximilians-Universität (LMU), Munich, Germany; 9 German Center for Neurodegenerative Diseases, Munich, Germany; 10 Department of Neurology, RWTH Aachen University, Aachen, Germany; 11 JARA-BRAIN Institute Molecular Neuroscience and Neuroimaging, Research Centre Juelich GmbH, Aachen, Germany; 12 Department of Neurology and Hertie-Institute for Clinical Brain Research, University of Tübingen, Tübingen, Germany; 13 Department of Neurology, Center for Movement Disorders and Neuromodulation, Medical Faculty, Düsseldorf, Germany; 14 Department of Health Research Methods, Evidence and Impact, McMaster University, Hamilton, Ontario Canada; 15 Department of Neuroradiology, University Hospital Bonn, Bonn, Germany

**Keywords:** Ataxia, Communication, Patient-related outcome measure, Speech, Hearing, Cerebellar cognitive affective syndrome

## Abstract

Background and Objectives: Friedreich ataxia (FA) causes progressive impairment of communication due to gradual deterioration of speech, associated with impaired hearing, socio-cognitive and language skills. There is an urgent need to investigate the impact of this multiparametric alteration on patients’ lives. Therefore, the COMunication and ATAXia measure (COMATAX) was developed and validated in French and German. Methods: In the PROFA study (NCT05943002), we conducted focus groups and cognitive interviews with patients with FA, professionals and caregivers to elaborate relevant items of communication disabilities. Subsequently, the measure was validated in 93 patients with FA via a mobile health app. For validation, distribution properties, reliability (Cronbach’s alpha) and validity (correlations with VHI-30, SSQ-12, SARA total score, SARA speech item, Speech rate and GAA repeats; known-groups validity by age, sex, and self-rated health/wellbeing, disease-severity, hearing function, daily activities, and exploratory factor analysis) were calculated. An IRT analysis was performed to assess item characteristics. Results: COMATAX consists of 17 items (five response options each) and an 18th question asking about the most bothersome symptom. We observed high internal consistency for the total COMATAX scale (α = 0.897), strong correlations with VHI-30 (*r*=.894), SSQ-12 (*r* = −.531), moderate with SARA score (*r*=.498), SARA speech item (*r*=.416), Speech rate (*r*=-.354), weak with *GAA* repeat length (r_*GAA1*_=-0.207) and the ability to distinguish between different subgroups (disease severity, self-rated health, wellbeing, hearing function, and daily activities). Most items showed good discrimination of communication ability. Conclusion: The COMATAX is a valid and reliable patient-reported communication disability measure, useful in future therapeutic trials in FA.

**Supplementary Information:**

The online version contains supplementary material available at 10.1007/s12311-026-02020-3.

## Introduction

Friedreich ataxia (FA) is a rare (1/50000) autosomal recessive neurological disease [1] and the most common hereditary cerebellar ataxia [2]. FA generally begins before the age of 20 and progresses gradually, with patients using a wheelchair on average 15 years after disease onset [3]. Patients present with a balance disorder (cerebellar and sensory ataxia), along with muscle weakness and scoliosis. This is associated with sensory loss, visual difficulties, and cardiomyopathy [2].

FA also affects the patient’s communication in multiple ways. Impaired speech and voice can be a result of damage to the cerebellar pathways, with imprecise consonant articulation, slower articulatory movements and speech rate. Patients also have difficulty regulating their speaking breath, resulting in shorter utterances [4]. The vocal timbre may be strained, hoarse, nasalized or unstable [5]. A considerable proportion of patients have auditory neuropathy [6, 7], which mainly implies difficulty understanding speech in noisy environments. Finally, certain features of Cerebellar Cognitive Affective Syndrome (CCAS) [8] include impairments in language, attention, executive functions, emotional regulation and social cognition [9], that could influence the quality of social interaction. This is accompanied by frequent visual disturbances [2], which could prevent lip-reading and decoding of written language. Additionally, patients have trouble activating automatic functions such as reading [10]. These communication impairments do massively affect the patients’ quality of life, making it highly necessary to measure the impact of these disorders on communication with reliable self-rated tools.

There are several domain-specific questionnaires for speech and hearing; however, their length and focus may limit their suitability for patients with FA. Widely used instruments such as the “Voice Handicap Index” [11] (VHI) and “Speech Handicap Index” [12] (SHI) (30 questions each) and the “Speech, Spatial and Qualities of Hearing Scale” [13] (SSQ) (49 questions) are lengthy and were not specifically designed for individuals with neurological conditions. Despite availability of short versions, they do not cover relevant FA specific domains of communication, such as lexical access, social cognition or processing speed.

To address the unmet need for a more suitable instrument covering the wide range of communication aspects, we aimed to develop and validate a questionnaire for self-assessment of communication disability specific to ataxia-related diseases as FA – COMATAX (COMmunication in ATAXia) – as part of the PROFA study [14].

## Methods

### Study Project and Sample

The Patient-reported, health economic and psychosocial outcomes in patients with Friedreich ataxia (PROFA) study (2022 to 2025) is a multicenter, prospective observational study conducted in France, Germany, and Austria, aiming to assess the medical, economic, and psychosocial impact of FA in daily life [14, 15]. Using momentary assessment via a mobile health (m-health) application (ATOM5 by Aparito), patients complete monthly self-reports over six months. Key objectives include evaluating the app’s usability, validating existing health and psychosocial measures, and developing and validating a new FA-specific communication questionnaire. The app is embedded within the Atom5 platform, allowing for the remote and digital capture of patient-generated data, including speech recordings [14]. The study will continue until 2028, with two additional follow-ups on month 12 and 18. Patients, aged ≥ 12 years with a diagnosed FA, an ataxia severity ≤ 30 Scale of Assessment and Rating of Ataxia (SARA) points and access to a digital device were eligible to participate.

### ClinicalTrials Registration

The PROFA study was prospectively registered (ClinicalTrials.gov NCT05943002 on July 12 2023).

### Development of COMATAX

The development and validation of COMATAX started with a qualitative phase (development) and followed by a quantitative phase (validation), considering scientific recommendations of the COnsensus-based Standards for the selection of health Measurement INstruments (COSMIN) [16]. The questionnaire was initially created in French language and subsequently translated into German.

#### Step 1: Collect patient, caregiver and professional input through qualitative research

To identify symptoms and impacts that matter most to patients and explore their language and descriptions, focus groups were conducted. After a comprehensive literature review, the most described topics related to communication difficulties in FA were listed and presented to the participants in an open list form (See Interview guide in eTable 1).

##### Participants

Three focus groups composed, respectively, of three persons with FA, six healthcare professionals specialized in FA and five caregivers (eTable 2). As part of the PROFA consortium, the French Friedreich Ataxia Association recruited individuals with FA and caregivers (convenience sample).

##### Procedure

Focus groups were all led by one speech therapist and two students (referred to as “evaluators”) in a semi-structured way. The focus group’s objective was to gather as many individuals as possible who have experienced communication difficulties related to FA. Each session ended when no participant had any further comments to add. All sessions were video recorded but only the audio was used for the transcription.

##### Data Analysis

Recordings were transcribed, including non-verbal auditory cues (e.g., laughter, hesitation). Each evaluator performed independently a thematic analysis with an inductive approach to coding for item generation. Then the results were pooled. 

#### Step 2: Content validity of the item list through cognitive interviews

Three cognitive interviews were performed by the evaluators to reduce discrepancies between item intent and participant interpretation and to identify ambiguities. A convenience sample of four participants were invited to participate remotely (eTable 3) where the technique of paraphrasing was employed (restate the item in own words) to ensure the meaning of each item. By this reformulation, the evaluators ensure that they agree with the participants on the meaning of each item. Subsequently, the evaluators asked the participants to select the most relevant items. Three criteria guided the final selection of statements: the items most favored by participants, those offering the simplest and understandable formulations, and those allowing to sweep the broad spectrum of communication difficulties in Friedreich Ataxia.

#### Step 3: Final item list and construct of the questionnaire

All choices and comments made by patients and caregivers were considered by the entire research team to create a final item list. To verify the clarity of the statements, the questionnaire was shared for rereading with three people who do not belong to the medical, paramedical or Friedreich community (lay participants without a professional background in healthcare, recruited from the social networks of the research team). The questionnaire COMATAX was constructed as a Likert scale based on item frequency. 

#### Step 4: Pretesting

Nine patients with FA (< 21 years old and able to work) were asked to complete the questionnaire in a paper version and comment on unclear items. 

#### Step 5: German-language translation

According to the Professional Society for Health Economics and Outcomes Research (ISPOR) recommendations [17], two bilingual individuals (one FA-specialist, one non-specialist) independently translated the French version into German. A third bilingual ataxia specialist synthesized both into one version and translated it back into French for content approval. The German version was validated by all three and revised by two monolingual German neurologists. No cognitive debriefing was conducted with German patients with FA, but the German Patient Advocacy Organization and German PROFA team members reviewed the version without changes. After finalization of the French and German COMATAX versions, the measure was integrated into the PROFA study. 

### Validation of COMATAX

#### Measures and Data Assessment

The COMATAX was assessed at baseline (in the French study center) and as a digital version (from patients from all study sites) via the mobile health app subsequently after the study center baseline assessment. For validation, the app-based COMATAX responses were used together with the following additional data of the PROFA study:

#### Sociodemographic and Genetic Data

Sociodemographic data includes age, sex, education and age of onset. Genetic data consists of *Guanine-Adenine-Adenine* (*GAA)* repeat length of the shorter allele *GAA1* and the mean of both *GAA1* and *GAA2*.

#### Self-Rated Voice and Hearing Measures

The Voice-handicap Index (VHI) [11] (30 items, score range 0 to 120, measure of voice) and the Speech, Spatial, and Qualities of Hearing measure [18] (SSQ-12, short-form, 12 items, score range 0 to 10, auditory disorders measure) were used as construct-related measures.

#### Self-Rated Health-Related Measures

Health-related measures include the activities of daily living as part of the Friedreich Ataxia Rating Scale [19] (ADL-FARS, nine items, score range 0 to 36), the disease-specific patient-reported impairment measure PROM-Ataxia Short Form [20] (10 items, score range 0 to 40), and the Warwick-Edinburgh Mental Well-being Scale [21] (WEMWBS, 14 items, score range 14 to 70).

#### Performance-Related Measures

The Scale of Assessment and Rating of Ataxia (SARA) [22] for ataxia severity was rated by a clinician. SARA is made up of 8 items related to gait (/8), stance (/6), sitting (/4), speech disturbance (/6), finger-chase test (/4), nose-finger test (/4), fast alternating movements (/4) and heel-shin test (/4), with a total score varying from 0 to 40. The higher the score, the more severe the ataxia. The SARA total score and the speech item score were included in the validation analyses. Auditory screening was performed with “digit in noise” test to assess if the patient is at risk for hearing loss [23]. To assess the occurrence of Cerebellar Cognitive Affective Syndrome (CCAS), the CCAS scale was used (10 tests), classified as no (no failed test), possible (1 failed), probable (2 failed) or definite CCAS (≥ 3 failed) [24]. Additionally, Speech rate was recorded. Patients were asked to repeat the day of the week for 30 s via the Atom5 app, approximately eight days after baseline. Due to the variable length of the records, only the first 20 s were analyzed. To determine the speech rate per second, the total number of phonemes pronounced was summed and divided by 20. The lower the number of phonemes per second, the slower the rate of speech, suggesting greater dysarthria.

### Statistical Analysis

#### Distribution of item and score values

Frequency of each item response option as well as mean, standard deviation, range, median, skewness and kurtosis for the COMATAX score were calculated, visualized with kernel-density plots and tested for normal distribution by Kolmogorov-Smirnov test.

#### Validity

Correlation analyses were conducted between COMATAX, VHI-30, SSQ-12, SARA total score, SARA speech score, Speech rate and *GAA* (short and *GAA1* and *GAA2* mean), using the Spearman correlation coefficient (r_p_), interpreted as follows: r_sp_<0.1 = small, r_sp_ 0.3 ≥ r_sp_<0.5 = moderate and r_sp_ ≥ 0.5 r_sp_=strong [25]. VHI-30 and SSQ-12 were expected to correlate similarly strongly with COMATAX as these instruments capture related patient-perceived constructs of communication, hearing and speech. In contrast, lower, but moderate correlations were expected with SARA total score, SARA speech score, speech rate and *GAA* repeats as these reflect clinician-rated or objective outcomes that differ from conceptually patient-reported perspectives. The COMATAX’s ability to distinguish between different groups was analysed using the concept of *known-groups* validity, expecting differences in disease severity (SARA_meansplit_: ≤16 = low;>16 = high), self-rated health (PROMATAX_meansplit_: low/high), well-being (WEMWBS_meansplit_: low/high), activities of daily living (FARS-ADL_meansplit_: low/high), disease onset (early/late), hearing impairments (Digit in noise no problems/problems) and CCAS (normal to possible/probable to definite). No differences were expected for education level (International Standard Classification of Education (ISCED): low/moderate/high), sex (female/male) and age_meansplit_ (≤ 34 and > 34 years old). T-test for two groups and ANOVA for more than two categories with effect sizes (Cohens d for t-test and *η²* for ANOVA) were used. Additionally, exploratory subgroup analyses were conducted to assess potential differences in COMATAX scores between children and adults and between individuals with late-onset FA (LOFA) and earlier onset using the Mann–Whitney U test. To explore the COMATAX factor structure, an *exploratory factor analysis (efa)* was performed using the eigenvalue approach and scree plot to determine the number of factors, followed by maximum likelihood analysis with oblimin rotation. Items with factor loadings < 0.3 were excluded. The suitability of efa was confirmed by the Kaiser-Meyer-Olkin (KMO) measure and the Bartlett’s test. If necessary, items were classified to a factor by the researchers’ best knowledge. 

#### Reliability

Internal consistency of the COMATAX scale and for the proposed factors of the efa was tested using Cronbach’s alpha [26], with α ≥ 0.7 interpreted as acceptable, α ≥ 0.8 as good and α ≥ 0.9 as excellent. For test-retest reliability of the COMATAX, we calculated the Intraclass Correlation Coefficient (ICC) between scores obtained at baseline and those from the first app-based assessment two weeks after baseline (exists only for French patients), expecting high agreement. ICCs were computed with a two-way mixed-effects model with absolute agreement, with ICC > 0.9 indicating excellent, > 0.75 good, > 0.5 moderate and < 0.5 poor agreement [27].

#### IRT for Item Characteristics

The Graded Response Model (GRM) was applied to evaluate item-level characteristics of the COMATAX scale. Prior to applying the GRM, unidimensionality was tested and considered sufficient with > 40% explained variance by a single factor. Two item parameters were estimated: the discrimination parameter (α) and the difficulty thresholds (b1–b4). The α parameter reflects how well an item differentiates between individuals across the latent trait continuum, with steeper slopes indicating higher discriminative power. Item discrimination was classified according to Baker and Kim with None: 0; Very low: 0.01–0.34; Low: 0.35–0.64; Moderate: 0.65–1.34; High: 1.35–1.69; Very high: ≥1.70 [28]. Threshold parameters indicate the points on the latent trait (θ) at which a respondent has a 50% chance of selecting a given response category or a higher one, reflecting the difficulty of transitioning between adjacent categories. Item characteristic curves were generated for visualization.

Statistical analyses were performed using IBM SPSS and the R package *mirt* (version 1.32.1) for IRT.

## Results

### Development of COMATAX 

#### Step 1: Collect patient, caregiver and professional input through qualitative research

Based on the focus groups, 87 items were created and sorted into 16 different topics (Table 1). Healthcare professionals reported dysarthria and dysphonia as the most troublesome communication disabilities in FA, whereas patients and caregivers pointed to difficulties such as managing breath and swallowing when speaking, the fatigue that communicating causes (not only in speaking but also in perceiving, understanding and finding the words to respond), and the inability to communicate while multitasking, especially when walking. In terms of emotions, carers reported reactions that are sometimes inappropriate in context and intensity. Patients reported that they feel emotions more strongly than they did before FA onset (Table 1). For the cognitive interviews, this list was reduced to 74 items grouped into 8 domains: speech (*n* = 15), hearing (*n* = 3), language (*n* = 6), cognition (*n* = 10), emotions (*n* = 12), effort and fatigue (*n* = 6), psychological impact (*n* = 13), autonomy and communication strategies (*n* = 9).

**Table 1:** A summary of the themes listed by evaluators. Example of items of each topic

Topics	Example of items
1) Speech Motor Control	“There’s a gap between what I’d like to say and the result.” “I have a numbness in my mouth when speaking.”
2) Coordination of speech and swallow	“I have difficulty swallowing when I speak.” “My speech is affected when I swallow my saliva.”
3) Coordination of speech and breath	“When I speak, my breath and my voice are not in phase.” “I sometimes cut off in the middle of a word because I’m out of breath.”
4) Voice loudness	“I wonder if my voice can be heard.” “My voice is not strong.”
5) Hearing in noise	“I find it challenging to follow conversations with multiple people.” “I have difficulty understanding speech in noise.”
6) Hearing in quiet	“I must listen carefully to hear properly, even in silence.”
7) Written language (reading and writing)	“I have the ability to communicate through writing (through text message, email, or pen and paper).”
8) Lexical access	“It’s difficult for me to find my words.” “I need time to find my words.”
9) Speed processing and rhythm	“When I am asked a question, I require time to formulate my response.” “I intervene with a delay in the conversation.”
10) Attention and dual task	“I must concentrate to speak.” “I need to avoid multitasking when speaking.”
11) Emotional decoding	“At times, I feel out of touch with those around me when it comes to emotions.” “I have trouble understanding what others feel, think or want.”
12) Expressivity and emotional regulation	“I can’t control my emotions” “I’m often told that I’m not very expressive.”
13) Effort and fatigue	“Physical effort is necessary for me to communicate effectively.” “To speak properly, I must take a break during the day.”
14) Communication strategies	“I assign long or complex explanations to someone close to me.” “I try to save time before answering a question so that I can work out my answer.”
15) Psychosocial impact	“I’m afraid of how others will look at me when I communicate.” “I’m frustrated/thrilled/angry at having trouble communicating.”
16) Autonomy and social life	“I find it hard to make myself understood by people I don’t know.” “My communication skills limit the creation and/or maintenance of friendly or professional relationships.”

#### Step 2: Content validity of item list through cognitive interviews

The interviews led to various item reformulations or clarifications. Certain items emerged as being more important to some participants than to others (Tables S3). The evaluators then synthesized the interview data to select the final set of 17 items, forming the first version of the scale.  

#### Step 3: Final item list and construct of the questionnaire

Minor rewording was applied to two items to improve comprehensibility: “My articulation is imprecise” was revised to “I find it hard to articulate”, and “I have to pay attention so that I understand in the silence” was rephrased as “I can’t hear, even in quiet places.” One item (“I have to stop talking to swallow my saliva”) was removed due to redundancy, while two new items were added based on expert feedback and patient relevance: “I speak less well when I’m tired” and “I speak slowly.”

The final questionnaire consists of 17 items rated on a 5-point-Likert scale (0 = never to 4 = always) based on experienced symptom frequency over the past two weeks, as frequency was easier for patients to recall than symptom severity and to be in line with other questionnaires used in PROFA. The clinical and research team decided to add an 18th item captures the patient’s most bothersome symptom (MBS) to address individual variability. From 0 to 68, the higher the score, the greater the difficulties. 

#### Step 4: Pretesting

The questionnaire was well accepted by the participants and completed within 10 min. Only two responses were missing, both from the same participant. One item has generated requests for clarification: “I feel strong emotions”, which was reworded in “I feel emotions more strongly”.

#### Step 5: Translation

The German version has the same construction, consisting of 18 items and a 5-point Likert scale. Only minor changes to the wording of items n°6, 8, 10, 11, 15, and 18 were made. 

### Validation of COMATAX

#### Sample

We included *n* = 93 patients (French-speaking patients: *n* = 50; German-speaking patients: *n* = 43) in our analysis. Sample characteristics are presented in Table 2. The mean age was 35.9years (by language_French/German_: 36.4/35.2); with nearly half of the participants being female (total 51.6%; French-speaking: 58.0%/German-speaking: 44.2%). The mean age at onset was 19 years. For the total sample, disease severity measured by the SARA score was 17.4 points (19.0/15.5, *p* =.006).

**Table 2:** Description of the sample

	Total (*n* = 93)	French speaking patients (*n* = 50)	German speaking patients (*n* = 43)	*p*-value
Sociodemographic variables				
Sex, female %	51.6	58.0	44.2	0.262
Age, M ± SD, range, years	35.9 ± 14.8; 14–69	36.4 ± 13.6; 15–69	35.2 ± 16.2; 14–67	0.701
*GAA1* (short allele) *n* = 77 (French *n* = 43; German *n* = 34) Mean of both alleles^§^ *n* = 70 (French *n* = 40; German *n* = 30)	517.0 ± 289.3 649.0 ± 302.5	501.3 ± 288.8 673.9 ± 326.3	536.8 ± 293.0 616.7 ± 270.5	0.596 0.433
Marital status, % Single Married/living with partner Other (widowed, divorced, separated) Having children %	44.1 46.2 9.7 37.6	34.0 52.0 14.0 50.0	55.8 39.5 4.7 23.3	0.031 0.015
Education level, % low medium high	17.2 37.6 44.1	12.0 40.0 48.0	23.3 34.9 39.5	0.328
Employment, %	50.0	42.0	55.8	0.262
Disease-related variables				
Age at onset, M ± SD years	19.1 ± 11.7	19.5 ± 10.6	18.6 ± 12.9	0.487
SARA score, M ± SD/40	17.4 ± 5.9	19.0 ± 5.9	15.5 ± 5.7	0.012
Disability stages, % No handicap Mild Moderate Severe Walking with sticks Unable to walk Confined to bed	-- 5.4 29.0 10.8 31.2 22.6 --	-- 2.0 18.0 14.0 32.0 34.0 --	-- 9.3 41.9 7.0 30.2 9.3 --	0.007
Speech rate	7.57 ± 2.1	6.82 ± 1.6	8.51 ± 2.2	0.000
Self-rated health outcomes				
COMATAX score, M ± SD	23.4 ± 12.0	25.8 ± 12.6	20.7 ± 10.8	0.040
VHI-30 score*, M ± SD	32.0 ± 23.5	37.9 ± 25.7	24.3 ± 17.9	0.028
SSQ-12 score*, M ± SD	7.8 ± 2.0	7.6 ± 2.3	8.0 ± 1.5	0.404
FARS-ADL, M ± SD	12.5 ± 4.8	12.9 ± 5.3	12.0 ± 4.3	0.381
PROM-ATAX, M ± SD	20.1 ± 8.1	21.7 ± 8.2	18.2 ± 7.6	0.036
WEMBWBS, M ± SD	49.8 ± 9.0	48.9 ± 8.6	50.7 ± 9.5	0.369
HearWHO, % Hearing problems	40.9	54.0	23.7	0.000
CCAS (failed tasks) 0 (normal) 1 (possible CCAS) 2 (probable CCAS) ≥ 3 (definite CCAS)	16 17 13 42	7 6 5 30	9 11 8 12	0.024

#### Distribution of Items and Score Values

**Fig. 1:**
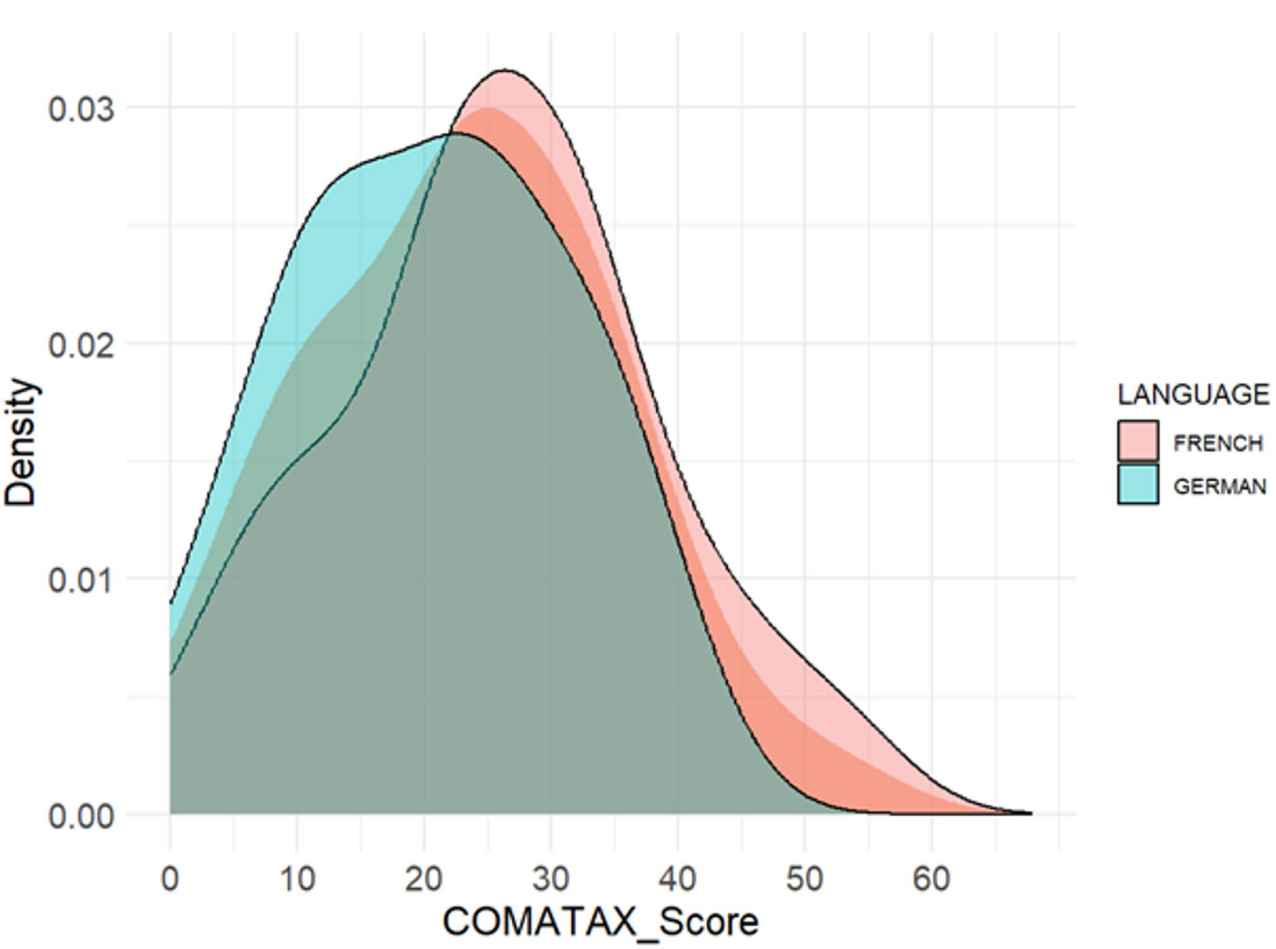
Kernel-density plot of COMATAX score overall and by language. *Note.* COMATAX score can be ranged between 0 and 68. The shadowed distribution visualized the total sample. The distribution of the German-speaking individuals is slightly more right-skewed, suggesting that most participants experienced lower communication difficulties compared to the French-speaking individuals

There is a varying distribution across items (Table 3), with some items showing higher concentration of responses in extreme categories (e.g., item 11: never = 68.8%) compared to others with higher proportion of the mid-response option “rare” (e.g., item 1 = 44.1%; item 4 = 34.4%). One patient reported no problems with each item. The mean COMATAX score was higher in the French (25.8 ± 12.7) compared to the German group (20.7 ± 10.8). Both groups exhibit approximately symmetric distributions, with the German group tending to be flatter. Kolmogorov-Smirnov tests were non-significant for both groups, indicating normality. Figure 1 illustrates kernel density plots, showing higher density in lower scores for the German-speaking group and higher scores for the French-speaking group. The most frequently chosen bothersome items were “difficult articulation” (item 1) followed by “understanding difficult in noise” (item 10).

**Table 3:** Distribution properties and frequency of each response option

	*n*	mean ± SD	min	max	median	skewness	kurtosis
COMATAX total	93	23.4 ± 12.1	0	54	24	0.13	−0.44
COMATAX French	50	25.8 ± 12.7	0	54	25.5	0.07	−0.41
COMATAX German	43	20.7 ± 10.8	1	39	22	−0.02	−1.08
Items	**Never**	**Sometimes**	**Rare**	**Often**	**Always**	**Floor effect**,** %**	**Ceiling effect**,** %**
Item 1 difficult articulation	8	26	**41**	14	4	8.6	4.3
Item 2 slow speech	14	13	20	**27**	19	15.1	20.4
Item 3 weak voice	23	21	**30**	16	3	24.7	3.2
Item 4 shortness of speaking breath	21	29	**32**	9	2	22.6	2.2
Item 5 gives up long sentences	**33**	26	24	9	1	35.5	1.1
Item 6 group discussions too fast	**39**	22	16	15	1	41.9	1.1
Item 7 difficulty in finding words	28	**41**	14	8	2	30.1	2.2
Item 8 difficulty in dual task with speech	23	18	**28**	17	7	24.7	7.5
Item 9 speech more difficult when tired	10	10	20	**29**	24	10.8	**25.8**
Item 10 understanding difficult in noise	20	23	**24**	19	7	21.5	7.5
Item 11 hearing difficult in quiet	**64**	24	3	2	0	**68.8**	0.0
Item 12 written communication difficult	**47**	22	13	8	3	50.5	3.2
Item 13 emotional reactions different from others	**43**	21	18	10	1	46.2	1.1
Item 14 emotions felt more strongly	**31**	22	16	17	7	33.3	7.25
Item 15 friendships difficult	**55**	22	4	9	3	59.1	3.2
Item 16 trouble accepting my speech	**30**	20	21	14	8	32.3	8.6
Item 17 tiring communication	27	**26**	19	13	8	29.0	8.6

*Note.* n, number of observations, SD, Standard Deviation; Response frequency: in bold= most common answer option; Ceiling and floor effect: in bold= Item with the highest amount of ceiling and floor effect

#### Validity

COMATAX correlated strongly with VHI-30 (*r*=.894, *p* <.01), SSQ-12 (*r*=-.531, *p* <.01), moderately with SARA (*r*=.498, *p* <.01), SARA speech (*r*=.416, *p* <.01), Speech rate (*r*=.354, *p* <.01) and weakly with *GAA* (r_*GAA*short_=−0.207, *p* <.07; r_*GAA*mean_=−0.136, *p* =.257). Subgroup analyses yielded similar patterns, with higher correlations with SSQ-12, SARA score, SARA speech and Speech rate and lower correlations with *GAA* in the French sample (eTable 4).

Results, shown in Table 4, supported the assumptions regarding differences between known groups. COMATAX scores were higher in individuals with higher disease severity (SARA), lower self-rated health (PROM-Ataxia), reduced wellbeing (WEMBWBS), hearing impairments (HearWHO), and in patients with more limitations in ADL (FARS-ADL). No differences were found according to disease onset, CCAS, educational level, sex and age. Furthermore, exploratory subgroup analyses showed no statistically significant differences in COMATAX scores between children (*n* = 11) and adults (*n* = 82) or between individuals with late-onset FA (*n* = 66) and earlier onset (*n* = 27).

**Table 4:** Known-groups validity of COMATAX

Sub groups	COMATAX Score (mean ± SD)	*n*	*p*-value	Effect size (^a^Cohens d/^b^η²)
SARA Score				
Low (≤ 16)	18.0 ± 11.3	47	< 0.01	1.021^a^
High (> 16)	29.1 ± 10.3	45		
PROM-Ataxia Short Form				
Low (≤ 22)	18.5 ± 10.3	54	< 0.01	1.127^a^
High (> 22)	30.3 ± 10.8	39		
WEMBWBS				
Low (≤ 50.5)	27.0 ± 12.3	41	< 0.01	0.623^a^
High (> 50.5)	19.7 ± 11.2	45		
FARS-ADL				
Low (≤ 12)	18.1 ± 10.6	49	< 0.01	1.077^a^
High (> 12)	29.6 ± 10.8	43		
Disease onset				
≤ 15	23.2 ± 13.7	55	0.850	0.041^a^
> 15	23.7 ± 9.3	37		
Education				
1 (low education)	18.6 ± 10.7	16	0.201	0.040^b^
2 (medium education)	24.9 ± 12.3	35		
3 (high education)	24.1 ± 12.2	44		
Hear WHO				
No hearing problems	19.9 ± 11.1	52	< 0.01	0.820^a^
Hearing problems	29.0 ± 11.3	36		
CCAS				
Normal to possible (0 to 1 failed test)	20.6 ± 11.2	33	0.143	0.325^a^
Probable to definite (≥ 2 failed test)	24.4 ± 12.0	55		
Sex				
Female	25.8 ± 13.2	48	0.053	0.407^a^
Male	21.0 ± 10.2	45		
Age (years)				
≤ 34	21.4 ± 13.7	49	0.089	0.359^a^
> 34	25.7 ± 9.6	43		

Note. n, number of patients; SD, Standard Deviation, COMATAX, Communication in ataxia; SARA, Scale for the Assessment and Rating of Ataxia; WEMWEBS, Warwick-Edinburgh Mental Well-being Scale; FARS-ADL, Friedreich’s Ataxia Rating Scale - Activities of Daily Living Subscale; Hear WHO, Auditory screening “digit in noise” test, CCAS, Cerebellar Cognitive Affective Syndrome Scale; Education based on International Standard Classification of Education; effect sizes: small (η²≥0.01; Cohens d ≥ 0.3), medium (η²≥0.06; Cohens d ≥ 0.5), and large (η²≥0.14; Cohens d ≥ 0.8)

For factor analysis, a three-factor solution was identified, explaining 50.0% variance. One item was assigned differently by the researchers than suggested by the efa. Finally, all items could be sorted to one of the three factors named as follows: “Speech and Communication” (11 items), “Conversation/Hearing” (3 items), “Feelings/Emotions” (3 items).

#### Reliability

Cronbach’s alpha for the total COMATAX scale indicated nearly excellent internal consistency (α = 0.897). According to the efa, the subscale “Speech and Communication” demonstrated good reliability (α = 0.895), whereas the others are acceptable (“Conversation/Hearing” α = 0.689 and “Feelings/Emotions” α = 0.674). The COMATAX test-retest reliability indicated a high agreement between baseline and first app-assessment two weeks later (ICC=0.870, 95% CI:0.781–0.924).

#### Item Response Theory Test (IRT)

eTable 5 shows GRM item parameters from the IRT analysis. Discrimination (α) ranged from 0.690 to 3.177 (mean = 1.693). Items 13–14 have low discrimination, indicating limited differentiation. Difficulty thresholds (b1–b4) reflect ability levels needed for response category shifts; higher b-values – as seen in item 13 (b4 = 6.352) and item 14 (b4 = 3.957) – signal more challenging items. The item characteristic curves illustrate the probability of selecting a particular response category based on ability level (θ) (eFigure 1). Steeply rising curves indicate well-discriminating items, while flatter or overlapping curves, as seen in items 13, 14 and 11, suggest poorer differentiation of the latent trait.

## Discussion

This paper describes the development and validation of COMATAX, a novel patient-reported measure specifically designed to assess communication disabilities in individuals with FA, as part of the PROFA study [14]. The development of COMATAX was based on input from individuals with FA as well as caregivers and health professionals, ensuring both relevance and comprehensiveness. The final version of COMATAX comprises 18 items with a two-week recall period: 17 items address distinct aspects of communication disability in FA, and the final item captures the most bothersome symptom as perceived by the patient. By completing all items, a total communication disability score (ranging from 0 to 68) can be derived, reflecting the patient’s perception of their difficulties with higher scores represent more communication impairment. The validation findings support COMATAX as a reliable and valid measure for self-perceived communication impairments in FA.

It is important to incorporate patient perspectives in FA research, reflected in the development of Patient-Reported Outcomes Measures (PROMs) for this population. This aligns with the FDA’s [29] and the EMA’s [30] recommendations to integrate PROMs in clinical trials, capturing more comprehensive aspects of the disease that matter most to patients. Communication difficulties are a common and highly impactful symptom in FA, affecting quality of life. However, no ataxia-related PROM [20, 31–33] has specifically targeted all the problems with communication in individuals with FA to date. COMATAX addresses this pressing need for a disease-specific tool for assessment of communication disability in FA. Disease-specific tools are more precise, more sensitive to detect health changes and correlate higher with functional disease measures [34].

The validation results provide preliminary evidence that COMATAX is a reliable instrument for assessing self-reported communication impairments in people with FA. Distribution of COMATAX scores differed between the language groups, with slightly higher communication impairments in French-speaking patients. This aligns with the ataxia severity and the self-reported health status in each group, supporting the discriminative capacity of COMATAX. Overall, the respondents’ answers to each item were chosen more frequently at the first three response levels, indicating that the originally recruited sample was not affected by advanced disease, which aligns with the study inclusion criteria [14].

Significant strong correlations with VHI-30 [35, 36] for speech and SSQ-12 [18, 37] for hearing indicate adequate convergent validity of COMATAX. Lower but expected associations between COMATAX and SARA are likely caused by SARA’s broader, clinician-rated scope (speech plus mobility, balance and coordination) and underline the differing perspectives of clinician – vs. patient-reported measures [38–40]. Although there is a slight negative correlation between higher communication impairments (higher COMATAX score) and shorter *GAA1*, the low correlation score suggests that *GAA1* is not a strong predictor for self-perceived speech impairment in our sample. Self-perceived speech impairments in FA are likely influenced by multiple interacting factors beyond repeat length alone.

Known-groups validity confirmed COMATAX’s sensitivity in distinguishing subgroups by disease severity, self-rated health, well-being, hearing, and ADL. No statistical differences were observed between the CCAS groups. This finding may be explained by the fact that COMATAX is a multidimensional questionnaire capturing a broad range of communication-related domains, whereas the CCAS focuses on a partially set of abilities. The absence of significant subgroup differences between children and adults as well as late-onset and earlier onset of FA may be related to limited statistical power, particularly because of the small subgroups. IRT analysis supported its ability to differentiate communication impairments, with most items showing moderate to high discrimination. However, items 13 and 14 (emotion-related) showed limited relevance for patients with mild impairments, suggesting a need for further analysis in advanced FA stages or other ataxias (e.g., spinocerebellar ataxias) where emotional symptoms may be more prominent.

The efa revealed a three-factor structure with one item requiring manual allocation, possibly reflecting limited overlap between construct domains. Notably, only the factor “Speech and Communication” demonstrated good internal consistency, while the other two factors showed lower values, indicating a need for further item refinement or suggesting that COMATAX may primarily represent a unidimensional construct, what is underpinned by the high internal consistency, using all items.

### Strengths and Limitations

The rigorous, patient-centered development of COMATAX is a major strength. The qualitative approach actively involved individuals with FA and caregivers, ensuring representation of real-life experiences. The interdisciplinary development team, combining clinical and research expertise, further strengthened the design. However, some limitations must be noted. The qualitative sample predominantly consisted of individuals with late-onset Friedreich Ataxia, which may have influenced the wording and content of the developed questionnaire items and potentially led to an underrepresentation of more severe speech impairments. Furthermore, the number of individuals with FA included in the patient focus group was relatively small compared to the groups of healthcare professionals and caregivers. As a result, the patient perspective may be underrepresented in the initial identification of relevant communication difficulties. Moreover, cognitive debriefing was not conducted with German-speaking patients with FA, raising the risk of misinterpretation in this group. As development focused on French-speaking patients, cross-linguistic generalizability may be limited and thus could be influence the results of the COMATAX’s psychometric performance. With regard to the fact, that the study is going on, additional analysis with a bigger sample, separated by language group will be performed. Furthermore, cognitive interviews with German-speaking individuals with FA should be conducted. The 2-week recall period chosen for COMATAX may be considered a limitation. It was selected as a pragmatic balance between capturing a representative range of everyday communication situations and minimizing recall bias and respondent burden. Shorter windows (e.g., 24 h/7 days) may overemphasize daily fluctuations and miss infrequent but clinically relevant situations, whereas longer windows (≥ 4 weeks) may increase retrospective bias. Nevertheless, patient-centered investigations are needed to examine the appropriateness of the given recall period. Despite recruitment across six study sites, the validation sample was relatively small and larger samples are necessary to confirm the psychometric properties. Furthermore, the perspectives of children and adolescents were not explicitly considered during scale development, which may affect the appropriateness of the instrument for younger patients of the PROFA cohort. Additionally, participants of the PROFA study were mainly in less advanced disease stages (SARA total score < = 30), which makes it necessary to use the COMATAX in the future in patients with advanced ataxia severity (SARA total score > 30). While COMATAX was developed in individuals with FA, it should also be noted, that its conceptual focus on communication and speech may be applicable to other ataxias. Nevertheless, differences in disease characteristics across ataxia types may influence the relevance and interpretation of specific items, indicating that further validation and potential adaptation are required before broader use. Lastly, the instrument’s ability to detect communication changes over time requires further research.

## Conclusion

This study presents the development and validation of COMATAX, a disease-specific, self-reported instrument to assess communication disabilities in FA. The results provide initial support for COMATAX as a reliable and valid PROM tailored to the communication challenges in FA. However, certain items show potential for refinement to improve psychometric performance, which should be addressed across different disease stages and further ataxia populations.

## Supplementary Information

Below is the link to the electronic supplementary material.

Supplementary Material 1 (DOCX 84.3 KB)

## Data Availability

The data that support the ﬁndings of this study are available on request from the corresponding author. The data are not publicly available due to privacy or ethical restrictions.

## References

[1] Durr A, Cossee M, Agid Y, Campuzano V, Mignard C, Penet C, et al. Clinical and genetic abnormalities in patients with Friedreich’s ataxia. N Engl J Med. 1996;335(16):1169–75. 8815938 10.1056/NEJM199610173351601

[2] Reetz K, Lischewski SA, Dogan I, Didszun C, Pishnamaz M, Konrad K, et al. Friedreich’s ataxia-a rare multisystem disease. Lancet Neurol. 2025;24(7):614–24. 10.1016/s1474-4422(25)00175-9. 40541211 10.1016/S1474-4422(25)00175-9

[3] Pandolfo M. Friedreich ataxia: the clinical picture. J Neurol. 2009;256:3–8. 19283344 10.1007/s00415-009-1002-3

[4] Folker JE, Murdoch BE, Rosen KM, Cahill LM, Delatycki MB, Corben LA, et al. Differentiating profiles of speech impairments in Friedreich’s ataxia: a perceptual and instrumental approach. Int J Lang Commun Disord. 2012;47(1):65–76. 22268902 10.1111/j.1460-6984.2011.00078.x

[5] Vogel AP, Wardrop MI, Folker JE, Synofzik M, Corben LA, Delatycki MB, et al. Voice in Friedreich ataxia. J Voice. 2017;31(2):243.e9-243.e19. 10.1016/j.jvoice.2016.04.015 27501923 10.1016/j.jvoice.2016.04.015

[6] Rance G, Corben LA, Du Bourg E, King A, Delatycki MB. Successful treatment of auditory perceptual disorder in individuals with Friedreich ataxia. Neuroscience. 2010;171(2):552–5. 10.1016/j.neuroscience.2010.09.013. 20849937 10.1016/j.neuroscience.2010.09.013

[7] Giraudet F, Charles P, Mom T, Boespflug-Tanguy O, Dürr A, Deltenre P, et al. Rapid exhaustion of auditory neural conduction in a prototypical mitochondrial disease, Friedreich ataxia. Clin Neurophysiol. 2018;129(6):1121–9. 29625343 10.1016/j.clinph.2018.03.005

[8] Schmahmann JD, Sherman JC. The cerebellar cognitive affective syndrome. Brain. 1998;121(Pt 4):561–79. 10.1093/brain/121.4.561. 9577385 10.1093/brain/121.4.561

[9] Naeije G, Schulz JB, Corben LA. The cognitive profile of Friedreich ataxia: a systematic review and meta-analysis. BMC Neurol. 2022;22(1):97. 35300598 10.1186/s12883-022-02615-3 PMC8928653

[10] Sayah S, Rotgé J-Y, Francisque H, Gargiulo M, Czernecki V, Justo D, et al. Personality and neuropsychological profiles in Friedreich ataxia. Cerebellum. 2018;17:204–12. 29086357 10.1007/s12311-017-0890-5

[11] Jacobson BH, Johnson A, Grywalski C, Silbergleit A, Jacobson G, Benninger MS, et al. The voice handicap index (VHI) development and validation. Am J Speech Lang Pathol. 1997;6(3):66–70.

[12] Rinkel RN, V.d. Leeuw IM, van Reij EJ, Aaronson NK, Leemans CR. Speech handicap index in patients with oral and pharyngeal cancer: better understanding of patients’ complaints. Head Neck. 2008;30(7):868–74. 18302270 10.1002/hed.20795

[13] Gatehouse S, Noble W. The Speech, Spatial and Qualities of Hearing Scale (SSQ). Int J Audiol. 2004;43(2):85–99. 10.1080/14992020400050014. 15035561 10.1080/14992020400050014 PMC5593096

[14] Buchholz M, Weber N, Borel S, Sayah S, Xie F, Schulz JB, et al. Patient-reported, health economic and psychosocial outcomes in patients with Friedreich ataxia (PROFA): protocol of an observational study using momentary data assessments via mobile health app. BMJ Open. 2023;13(8):e075736. 10.1136/bmjopen-2023-075736. 37527887 10.1136/bmjopen-2023-075736 PMC10394552

[15] Grobe-Einsler M, Borel S, Buchholz M, Sayah S, Hilab R, Pierron L, et al. Patient-reported, psychosocial and health economic outcomes in mild to moderate Friedreich’s ataxia: baseline results of the PROFA study. Lancet Reg Health Eur. 2026;61:101552. 10.1016/j.lanepe.2025.101552. 41488489 10.1016/j.lanepe.2025.101552 PMC12756708

[16] Mokkink LB, Boers M, Van Der Vleuten C, Bouter L, Alonso J, Patrick DL, et al. COSMIN risk of bias tool to assess the quality of studies on reliability or measurement error of outcome measurement instruments: a Delphi study. BMC Med Res Methodol. 2020;20:1–13. 10.1186/s12874-020-01179-5 PMC7712525 33267819

[17] Wild D, Grove A, Martin M, Eremenco S, McElroy S, Verjee-Lorenz A, et al. Principles of good practice for the translation and cultural adaptation process for patient-reported outcomes (PRO) measures: report of the ISPOR task force for translation and cultural adaptation. Value Health. 2005;8(2):94–104. 15804318 10.1111/j.1524-4733.2005.04054.x

[18] Noble W, Jensen NS, Naylor G, Bhullar N, Akeroyd MA. A short form of the Speech, Spatial and Qualities of Hearing scale suitable for clinical use: the SSQ12. Int J Audiol. 2013;52(6):409–12. 10.3109/14992027.2013.781278. 23651462 10.3109/14992027.2013.781278 PMC3864780

[19] Fahey MC, Corben L, Collins V, Churchyard AJ, Delatycki MB. How is disease progress in Friedreich’s ataxia best measured? A study of four rating scales. J Neurol Neurosurg Psychiatry. 2007;78(4):411–3. 10.1136/jnnp.2006.096008. 17056635 10.1136/jnnp.2006.096008 PMC2077798

[20] Schmahmann JD, Pierce S, MacMore J, L’Italien GJ. Development and validation of a patient-reported outcome measure of ataxia. Mov Disord. 2021;36(10):2367–77. 10.1002/mds.28670. 34115419 10.1002/mds.28670

[21] Tennant R, Hiller L, Fishwick R, Platt S, Joseph S, Weich S, et al. The Warwick-Edinburgh Mental Well-being Scale (WEMWBS): development and UK validation. Health Qual Life Outcomes. 2007;5:63. 10.1186/1477-7525-5-63. 18042300 10.1186/1477-7525-5-63 PMC2222612

[22] Schmitz-Hübsch T, du Montcel ST, Baliko L, Berciano J, Boesch S, Depondt C, et al. Scale for the assessment and rating of ataxia: development of a new clinical scale. Neurology. 2006;66(11):1717–20. 10.1212/01.wnl.0000219042.60538.92. 16769946 10.1212/01.wnl.0000219042.60538.92

[23] Zokoll MA, Wagener KC, Brand T, Buschermöhle M, Kollmeier B. Internationally comparable screening tests for listening in noise in several European languages: the German digit triplet test as an optimization prototype. Int J Audiol. 2012;51(9):697–707. 10.3109/14992027.2012.690078. 22762202 10.3109/14992027.2012.690078

[24] Hoche F, Guell X, Vangel MG, Sherman JC, Schmahmann JD. The cerebellar cognitive affective/Schmahmann syndrome scale. Brain. 2018;141(1):248–70. 10.1093/brain/awx317. 29206893 10.1093/brain/awx317 PMC5837248

[25] Cohen J. Statistical power analysis for the behavioral sciences. Routledge; 2013.

[26] Cronbach LJ. Coefficient alpha and the internal structure of tests. Psychometrika. 1951;16(3):297–334.

[27] Koo TK, Li MY. A guideline of selecting and reporting intraclass correlation coefficients for reliability research. J Chiropr Med. 2016;15(2):155–63. 10.1016/j.jcm.2016.02.012. 27330520 10.1016/j.jcm.2016.02.012 PMC4913118

[28] Baker FB, Kim S-H. Item response theory: Parameter estimation techniques. CRC; 2004.

[29] Patrick DL, Burke LB, Powers JH, Scott JA, Rock EP, Dawisha S, O’Neill R, Kennedy DL. Patient-reported outcomes to support medical product labeling claims: FDA perspective. Value Health. 2007;10:S125–37. 17995471 10.1111/j.1524-4733.2007.00275.x

[30] Meregaglia M, Malandrini F, Angelini S, Ciani O. The assessment of patient-reported outcomes for the authorisation of medicines in Europe: a review of European public assessment reports from 2017 to 2022. Appl Health Econ Health Policy. 2023;21(6):925–35. 10.1007/s40258-023-00827-3. 37659000 10.1007/s40258-023-00827-3 PMC10627987

[31] Seabury J, Rosero S, Varma A, Weinstein J, Engebrecht C, Dilek N, et al. Friedreich’s ataxia-health index: development and validation of a novel disease-specific patient-reported outcome measure. Neurol Clin Pract. 2023;13(5):e200180. 37646046 10.1212/CPJ.0000000000200180 PMC10462051

[32] Cano SJ, Riazi A, Schapira AH, Cooper JM, Hobart JC. Friedreich’s ataxia impact scale: a new measure striving to provide the flexibility required by today’s studies. Mov Disord. 2009;24(7):984–92. 19224613 10.1002/mds.22420

[33] Smith SC, Hendriks AAJ, Cano SJ, Black N. Proxy reporting of health-related quality of life for people with dementia: a psychometric solution. Health Qual Life Outcomes. 2020;18(1):148. 10.1186/s12955-020-01396-y. 32448322 10.1186/s12955-020-01396-y PMC7245851

[34] Patrick DL, Deyo RA. Generic and disease-specific measures in assessing health status and quality of life. Med Care. 1989;27(3):S217–32. 2646490 10.1097/00005650-198903001-00018

[35] Nawka T, Wiesmann U, Gonnermann U. [Validation of the German version of the Voice Handicap Index]. HNO. 2003;51(11):921–30. 10.1007/s00106-003-0909-8. 14605713 10.1007/s00106-003-0909-8

[36] Woisard V, Bodin S, Puech M. [The Voice Handicap Index: impact of the translation in French on the validation]. Rev Laryngol Otol Rhinol (Bord). 2004;125(5):307–12. 15856833

[37] Wyss J, Mecklenburg DJ, Graham PL. Self-assessment of daily hearing function for implant recipients: a comparison of mean total scores for the Speech Spatial Qualities of Hearing Scale (SSQ49) with the SSQ12. Cochlear Implants Int. 2020;21(3):167–78. 10.1080/14670100.2019.1707993. 31887255 10.1080/14670100.2019.1707993

[38] Nielsen LM, Kirkegaard H, Østergaard LG, Bovbjerg K, Breinholt K, Maribo T. Comparison of self-reported and performance-based measures of functional ability in elderly patients in an emergency department: implications for selection of clinical outcome measures. BMC Geriatr. 2016;16(1):199. 10.1186/s12877-016-0376-1. 27899065 10.1186/s12877-016-0376-1 PMC5129645

[39] Hussain N, Alt Murphy M, Lundgren-Nilsson Å, Sunnerhagen KS. Relationship between self-reported and objectively measured manual ability varies during the first year post-stroke. Sci Rep. 2020;10(1):5093. 10.1038/s41598-020-61834-1. 32198393 10.1038/s41598-020-61834-1 PMC7083900

[40] Kiechle ES, Bailey SC, Hedlund LA, Viera AJ, Sheridan SL. Different measures, different outcomes? A systematic review of performance-based versus self-reported measures of health literacy and numeracy. J Gen Intern Med. 2015;30(10):1538–46. 10.1007/s11606-015-3288-4. 25917656 10.1007/s11606-015-3288-4 PMC4579206

