# Supplementary material for: A Patient-Reported Outcome Measure of Communication Difficulties in Friedreich Ataxia: COMATAX
Source: Cerebellum. 2026 May 23;25(4):85. doi: 10.1007/s12311-026-02020-3 (PMC13197451; doi:10.1007/s12311-026-02020-3)
Supplement: Supplementary file 1 — Supplementary Material 1 (DOCX 84.3 KB) [file 12311_2026_2020_MOESM1_ESM.docx]

A patient-reported outcome measure of communication difficulties in Friedreich Ataxia:

COMATAX.

Supplementary materials

eTable 1. Interview guide. The questions were adapted to each participant’s profile (persons with FA, caregivers, and professionals)

| Theme | First question | Probes |
| --- | --- | --- |
| Functional dimension | How do you communicate in your daily life? In several contexts (home, work, school, friendships, neighborhood). | Can you provide an example of a communication situation that is easy/difficult? |
| Emotional impact | How do you feel when you communicate? How do you feel when you talk to someone for the first time? | When don’t you understand what you're saying? Or that you don't understand? |
| Physical feeling | What do you feel physically when you speak or listen? | Do you feel effort, pain, or fatigue? |
| Hearing and speech comprehension | How do you understand speech in noise and in groups? | Do you experiment with difficulties? Do you use lip-reading? |
| Social cognition/language (Theory of mind, perception of emotions, communication strategies) | Are you comfortable understanding what the other person is saying or feeling? Do you sometimes feel like you're fumbling for words?  Can you express your emotions the way you want to? | Do your words or attitudes vary depending on the person you talk to (friend, teacher, doctor, neighbor...)? |
| Evolution and variability | Do your communications fluctuate? | How does it change over time? |
| Tips | Do you have any tips you use to make communication easier? |  |
| Most Bothersome Symptoms | If you think about the different symptoms of your disease, which are the three most bothersome? |  |

eTable 2. Description of the three focus groups and main results

| **FOCUS**  **GROUP** | **Participants** | **Duration** | **Format** | **Most frequently reported symptoms** |
| --- | --- | --- | --- | --- |
| **Professionals**  **(n=6)** | Neurologist (n=2), psychologist (n=1), clinical research assistant (n=2), occupational therapist (n=1), speech-therapist (n=1). All have a high level of experience with ataxic patients including patients with FA. | 1H33 | Hybrid | Dysarthria,  Dysphonia,  Emotion out of sync with other people |
| **Persons with FA**  **(n=3)** | 1 male (42 years old, onset at 25), 1 male (36 years old, onset at 30), 1 male (36 years old, onset at 25), with communication difficulties impacting their daily life but ability to walk preserved. | 1H00 | Remote | The relationship between breathiness and phonation/swallowing,  Fatigue,  Dual task,  Experiencing stronger emotions |
| **Caregivers**  **(n=5)** | 1 mother (adult, 35 y. with FA) 1 wife and 1 father (adult, 45 y. with FA), 1 father (adult, 29 y. with FA), 1 member of French Association of Friedreich Ataxia board. | 1H37 | Remote | The relationship between breathiness and phonation/swallowing,  Fatigue,  Dual task,  Emotion out of sync with other people |

eTable 3. Description of the cognitive interviews and main results

| **Cognitive interview** | **Participants** | **Modality** | **Number of items at the beginning** | **Number of items at the end** | **Main items retained** |
| --- | --- | --- | --- | --- | --- |
| 1 | 2 caregivers (1 mother, 1 father, not related) | Remote (around 1h15) | 87 | 87 | Lack of vocal intensity, fatigue, effort to speak, giving up on what they want to say. |
| 2 | 1 person with FA | Remote (around 1h15) | 87 | 21 | The interaction between breathiness and phonation or swallowing, slow speech, imprecise articulation, word finding difficulties, speech processing, dual tasks, giving up on what they want to say, shame, anxiety, emotional lability, difficulty interpreting other people's emotions. |
| 3 | 1 person with FA | Remote (around 45 min) | 87 | 21 | The interaction between breathiness and phonation or swallowing, slow speech, imprecise articulation, word finding difficulties, speech processing, dual tasks, written communication, auditory disorders, giving up on what they want to say, shame. |

eTable 4. Correlations of the COMATAX with VHI-30, SSQ-12, and SARA, SARA speech, Speech rate and GAA

|  | **COMATAX**  **(total sample)** | **n** | **p value** | **COMATAX**  **(French)** | **n** | **p value** | **COMATAX**  **(German)** | **n** | **p value** |
| --- | --- | --- | --- | --- | --- | --- | --- | --- | --- |
| **VHI-30** | 0.894 | 57 | <0.01 | 0.868 | 32 | <0.01 | 0.935 | 25 | <0.01 |
| **SSQ-12** | -0.531 | 58 | <0.01 | -0.577 | 32 | <0.01 | -0.477 | 26 | <0.05 |
| **SARA Score** | 0.498 | 93 | <0.01 | 0.631 | 50 | <0.01 | 0.272 | 43 | .0773 |
| **SARA speech** | 0.416 | 92 | <0.01 | 0.423 | 50 | <0.01 | 0.408 | 42 | <0.01 |
| **Speech rate** | -0.354 | 72 | <0.01 | -0.457 | 40 | <0.01 | -0.134 | 32 | 0.465 |
| ***GAA* short** | -0.207 | 77 | <0.07 | -0.095 | 43 | 0.546 | -0.330 | 34 | 0.057 |
| ***GAA* mean** | -0.136 | 71 | 0.257 | -0.029 | 40 | 0.988 | -0.329 | 31 | 0.070 |

Note. Data are given as Spearman correlation coefficient (r_sp_); n, number of observations; r_sp_< 0.1=small, r_sp_ 0.3 ≥ r_sp_ < 0.5 = moderate and r_sp_ ≥ 0.5 r_sp_ = high/strong; COMATAX, Communication in ataxia; VHI-30, Voice Handicap Index 30; SSQ-12, Speech, Spatial and Qualities of Hearing Scale 12; *GAA*, *GAA* short allele, *GAA* mean, mean of short and long allele.

eTable 5. Discrimination and Difficulty Parameters for the COMATAX

| **Items** | **α** | **b1** | **b2** | **b3** | **b4** |
| --- | --- | --- | --- | --- | --- |
| Item 1 | 2.313 | -1.775 | -0.363 | - 1.039 | 2.105 |
| Item 2 | 2.238 | -1.311 | -0.597 | 0.122 | 1.020 |
| Item 3 | 1.383 | -1.076 | -0.552 | 1.357 | 3.041 |
| Item 4 | 1.759 | -1.021 | 0.183 | 1.646 | 2.890 |
| Item 5 | 2.699 | -0.376 | 0.435 | 1.443 | 2.616 |
| Item 6 | 2.707 | -0.164 | 0.523 | 1.111 | 2.707 |
| Item 7 | 1.118 | -0.936 | 1.118 | 2.216 | **3.881** |
| Item 8 | 1.248 | -1.104 | -0.178 | 1.139 | 2.488 |
| Item 9 | 2.095 | -1.596 | -0.905 | -0.125 | -0.846 |
| Item 10 | 0.833 | -1.796 | -0.241 | 1.268 | 3.321 |
| Item 11 | 0.848 | 1.092 | 3.741 | 4.874 | Not chosen* |
| Item 12 | 1.327 | 0.031 | 1.062 | 1.965 | 3.090 |
| Item 13 | **0.752** | -0.182 | 1.251 | 2.987 | **6.352** |
| Item 14 | **0.690** | -1.034 | 0.573 | 1.795 | **3.957** |
| Item 15 | 1.843 | 0.349 | 1.273 | 1.517 | 2.536 |
| Item 16 | 1.757 | -0.578 | 0.189 | 1.010 | 1.906 |
| Item 17 | 3.177 | -0.559 | 0.223 | 0.823 | 1.520 |

*the last response option of item 11 was not chosen by the participants.

eFigure 1. Item characteristic curves of the 17 items of the COMATAX


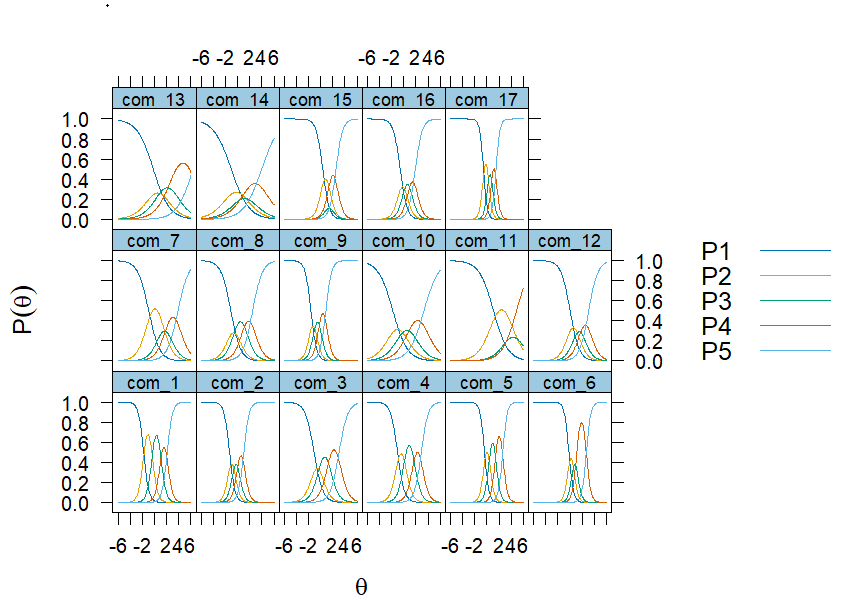


*Note*. com 1 to com 17: items from the COMATAX questionnaire 1 to 17; P: Probability (probability of a response category given a certain level of the latent trait θ; The latent trait represents the underlying ability or severity that is not directly observable but estimated from responses.)
